# Lipid Metabolism Modulation during SARS-CoV-2 Infection: A Spotlight on Extracellular Vesicles and Therapeutic Prospects

**DOI:** 10.3390/ijms25010640

**Published:** 2024-01-04

**Authors:** Heloisa D’Avila, Claudia Natércia Rocha Lima, Pollianne Garbero Rampinelli, Laiza Camila Oliveira Mateus, Renata Vieira de Sousa Silva, José Raimundo Correa, Patrícia Elaine de Almeida

**Affiliations:** 1 Cell Biology Laboratory, Department of Biology, Federal University of Juiz de Fora, Juiz de Fora 36036-900, Brazil; davila.bizarro@ufjf.br (H.D.); pollianneramp@hotmail.com (P.G.R.); laizacamila18@gmail.com (L.C.O.M.); renatisvieira@gmail.com (R.V.d.S.S.); 2 Laboratory of Microscopy and Microanalysis, University of Brasília, Brasília 70910-900, Brazil; claudianatercia@gmail.com

**Keywords:** extracellular vesicles, lipid bodies, COVID-19

## Abstract

Extracellular vesicles (EVs) have a significant impact on the pathophysiological processes associated with various diseases such as tumors, inflammation, and infection. They exhibit molecular, biochemical, and entry control characteristics similar to viral infections. Viruses, on the other hand, depend on host metabolic machineries to fulfill their biosynthetic requirements. Due to potential advantages such as biocompatibility, biodegradation, and efficient immune activation, EVs have emerged as potential therapeutic targets against the SARS-CoV-2 infection. Studies on COVID-19 patients have shown that they frequently have dysregulated lipid profiles, which are associated with an increased risk of severe repercussions. Lipid droplets (LDs) serve as organelles with significant roles in lipid metabolism and energy homeostasis as well as having a wide range of functions in infections. The down-modulation of lipids, such as sphingolipid ceramide and eicosanoids, or of the transcriptional factors involved in lipogenesis seem to inhibit the viral multiplication, suggesting their involvement in the virus replication and pathogenesis as well as highlighting their potential as targets for drug development. Hence, this review focuses on the role of modulation of lipid metabolism and EVs in the mechanism of immune system evasion during SARS-CoV-2 infection and explores the therapeutic potential of EVs as well as application for delivering therapeutic substances to mitigate viral infections.

## 1. Introduction

Respiratory viral diseases are a significant threat to human life and have been demonstrated to elevate pandemic risks, which is primarily due to their intricate transmission dynamics and viral evolutionary processes. In this context, the COVID-19 pandemic represented a significant global challenge. The etiological agent was identified as a novel coronavirus, named Severe Acute Respiratory Syndrome Coronavirus 2 (SARS-CoV-2), and the disease it causes was termed coronavirus disease 19 (COVID-19) [1]. It is now known that SARS-CoV-2 primarily spreads through respiratory droplets and aerosols generated by activities such as speaking, coughing, and sneezing [2,3,4,5].

The World Health Organization (WHO) has classified emerging SARS-CoV-2 variants into distinct categories based on their infectivity potential. Variants of Concern (VOCs) demand rapid attention, with specific VOCs (such as Alpha, Beta, and Gamma) under close surveillance [6]. Furthermore, the continuous emergence of the new SARS-CoV-2 variants/subvariants needs the perpetual vigilance of the healthcare domain and the scientific community to systematically observe the virus’s progression, pathogenic potential, virulence and transmissive attributes across different countries [7].

Currently, there are two VOCs in circulation: Delta and Omicron. Notably, the Omicron variant surpasses Delta in terms of resistance to antibodies [6]. To provide protection and manage the spread of the SARS-CoV-2 virus, the development of a vaccine became imperative. While the vaccine shields the recipient from developing severe illness, there are currently no available data indicating that the vaccine hinders the ongoing transmission of the virus. In addition, vaccine efficacy can be also hindered by a compromised host immune response [8,9,10].

Viruses and their hosts have undergone coevolution over an extended period, resulting in selective pressure on both the pathogen and the human immune system. On one hand, the immune system has adapted to counter viruses and infected cells, while viruses have developed intricate mechanisms to evade the immune response [11,12,13,14,15,16]. These pathogens manipulate host lipid metabolism to evade the immune system, promote their replication or trigger pathological responses [17,18]. Focusing on the interplay between SARS-CoV-2 and host lipid metabolism shows potential for the development of therapeutic strategies [17]. Despite the utilization of cellular organelles like lipid droplets (LDs), viral proteins, either independently or in concert with hijacked host proteins, swiftly alter the protein and lipid composition, as well as the structure, of LDs to achieve optimal conditions for replication [19]. Moreover, following replication and the assembly of new viral particles, (+) ssRNA viruses can commandeer membranes as carriers for non-lytic cellular exit, facilitating transmission to other susceptible hosts [19].

SARS-CoV-2 infection induces the formation of cytoplasmic membrane-bound vacuoles in respiratory epithelial cells, which are able to carry on virus-like particles. Both healthy and virally infected cells release EVs, which indeed serve as potential targets for the mediators of viral infection [20,21,22]. The action of EVs is mostly associated with their content, dependent on the state of homeostasis, as well as the cells of origin [23]. EVs are membrane-enclosed structures containing various biomolecules such as proteins, miRNAs, mRNAs, long non-coding RNAs (lncRNAs), DNA strands, lipids, and carbohydrates derived from their parent cells. Thus, they are capable of delivering these biomolecules to target cells, thereby reprogramming their metabolism, function, and morphology [24,25,26].

Increasing evidence underscores the crucial roles of secreted vesicles in the pathogenesis of several human diseases, notably cancer, neurodegenerative disorders and infections. The possible role of the EVs in incorporating pathogen-derived materials and becoming delivery vectors was described by Gould et al. in 2003 as the “Trojan exosome hypothesis” and was adapted in 2014 by Izquierdo-Useros N [27,28]. Over the past 40 years, scientists have explored the relationship between viral pathogenesis and exosomes [29,30]. In recent years, substantial evidence has emerged demonstrating the ability of EVs to transport and deliver viral genomes into recipient cells in vitro, as exemplified by studies involving Herpes Simplex virus 1 [31], Hepatitis C virus (HCV) [32], Hepatitis A virus (HAV) [33], Human Herpesvirus 6 (HHV-6) [34], and SARS-CoV-2 [35,36,37,38,39,40,41,42].

**Figure 1:**
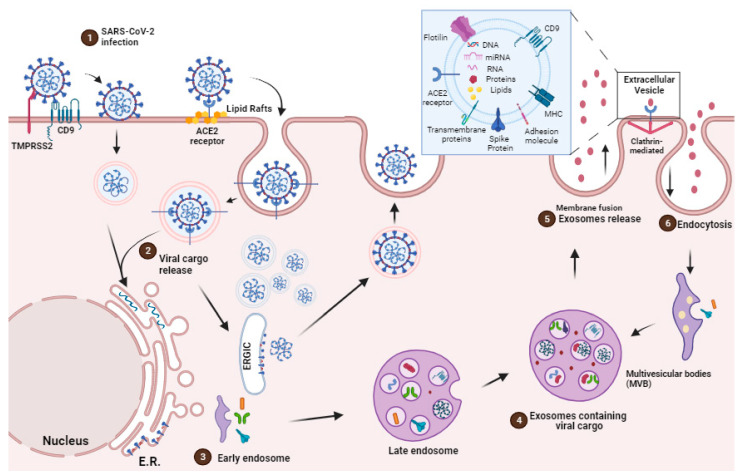
The SARS-CoV-2 infection cycle and the production of EVs. There are two distinct SARS-CoV-2 entry pathways. (1) CD9 works with TMPRSS2 to cleave viral fusion glycoproteins, facilitating coronavirus entrance. Alternatively, the virus binds to the ACE2 receptor in the lipid rafts region, where the virus–ACE2 complex is internalized via endocytosis. (2) The infection with a coronavirus causes the creation of new membrane structures of various sizes and forms in the perinuclear area, which are collectively referred to as replication organelles. Viral structural proteins and genomic RNA are produced at the replication site and subsequently translocated to the ER-Golgi intermediate compartment (ERGIC), where virus assembly and budding occur via an unknown mechanism. New viruses from the ERGIC lumen bud into the secretory route and reach the plasma membrane, where they are released into the extracellular environment after virus-containing vesicles fuse with the plasma membrane. In parallel, (3) SARS-CoV-2 arrives at lysosomes via the late endosome and (4) multivesicular bodies (MVBs), forming exosomes containing viral cargo (5), which are released into the extracellular environment. In parallel, EVs can be internalized by clathrin-mediated endocytosis (6). In detail, EVs carry proteins such as MHC, Tetraspanin, ACE2 and flotillin on their membrane while also containing viral and host cell components internally.

A prominent characteristic of SARS-CoV-2 infection involves the utilization of host cell membranes to generate replication organelles, exhibiting a spectrum of morphologies encompassing double-membrane structures and vesicular compartments. These organelles fulfill the function of safeguarding viral RNA from the host innate immune system sensors, thereby contributing to viral evasion mechanisms [38,39,40] (Figure 1).

Furthermore, EVs, serving as natural communication tools originating from living organisms, have garnered significant interest across diverse domains of biotechnology [43]. Their relevance is especially prominent in the field of medicine, where they are investigated for applications in disease diagnosis and treatment [42]. Significantly, EVs hold notable promise as vehicles for drug delivery and vaccine development. Thus, in this review, we provide a comprehensive overview of the impact of EVs in the host lipid metabolism, molecular mechanisms for EV-mediated cargo delivery and the advancements in potential therapeutics related to COVID-19 disease.

## 2. EVs Origins, Secretion and Communication

EVs are traditionally classified based on their size and mode of release from the cell. These vesicles are referred to by various names including exosomes, microvesicles (ectosomes), microparticles and apoptotic bodies (oncosomes). Exosomes are small EVs (40–100 nm) that bud from multivesicular bodies (MVBs) within cells and are released upon fusion of the MVBs with the cell plasma membrane, releasing their intraluminal vesicles in the extracellular environment in the form of exosomes [44,45,46,47]. 

EVs’ biogenesis is regulated by ceramide synthesis at the plasma membrane and endosomal levels [48,49]. The inhibition of neutral sphingomyelinase (nSMase) in both human and animal cells suppresses the release of exosomes while significantly boosting the release of microvesicles from the plasma membrane [49]. There are several proteins involved in exosome biogenesis such as endosomal sorting complex required for transport (ESCRT), vacuolar ATPase, and Vps4, which segregate and sort ubiquitylated proteins into intraluminal vesicles [8,24,50,51,52,53]. Menck et al. propose a novel regulatory function in vesicle budding from the plasma membrane and clearly suggest that despite the different vesicle biogenesis, the routes of vesicular export are adaptable [49].

Microvesicles or ectosomes, on the other hand, are larger EVs (200–500 nm) believed to bud directly from the plasma membrane [47,54,55]. Similar to exosomes, the generation of microvesicles involves the active participation of several protein factors. These factors encompass Ca^2+^-dependent aminophospholipid translocases, namely flippases and floppases, sphingomyelinase 2 (nSMase2), scramblases, and calpain. They collectively orchestrate the rearrangement of phospholipids, membrane curvature, and actin cytoskeleton reorganization, ultimately resulting in the extracellular formation of microvesicles [56,57,58,59]. Apoptotic bodies or oncosomes are fragments of cells undergoing programmed cell death, representing the largest EVs with a diameter ranging from 1 to 5 μm [47,55,56,60].

EVs from eukaryotic cells play a vital role in both local and systemic cellular communication, serving as intracellular mediators upon release into the extracellular space [26]. Since EVs have the same membrane orientation as cells, they expose on their surface the extracellular domains of transmembrane proteins that can bind to nearby or long-distance targets. These EVs transfer their bioactive cargo to target cells, thereby influencing and altering their behaviors [61]. Cell communication through EVs can occur through various mechanisms, including, paracrine, juxtacrine, endocrine or autocrine pathways [8]. The proper functioning of EVs relies on crucial steps, such as their release, uptake, and internalization [47,62].

The uptake of EVs is influenced by several factors, encompassing their dimensions, surface composition (lipids, glycans, proteins), pH conditions, temperature, and oxidative/hypoxic environment [8]. The nature of both the donor and recipient cells can modulate this process [63]. EVs are secreted through exocytosis or in multivesicular endosomes (MVEs), and they interact with target cells through ligand/receptor signaling at the plasma membrane [26]. After recognition, EVs can be taken up by the target cell through different pathways, such as macropinocytosis, phagocytosis, internalization via lipid rafts, clathrin-dependent endocytosis, caveolin-mediated uptake, and direct fusion of the EV membrane [26,64] (Figure 1). Once internalized, EVs follow the endocytic pathway with some undergoing recycling and re-secretion [64], while others may be directed to the lysosome for degradation [26]. Alternatively, EVs can fuse with the recipient cell plasma membrane, releasing their cargo directly into the cytosol [65]. 

The cargo of EVs, which includes lipids, DNA, RNA and proteins, can alter the transcription and signaling activity of recipient cells, thereby regulating their phenotype and function [8]. The protein composition of various types of EVs largely reflects that of the parent cells, exhibiting a notable enrichment of specific molecules. These include adhesion molecules, membrane-trafficking proteins, cytoskeleton components, heat-shock proteins, cytoplasmic enzymes, signal transduction molecules, cytokines, chemokines, proteinases, and cell-specific antigens [66]. They originate from both immune and non-immune cells, which play a vital role in immune system regulation. EVs also possess the capacity to either facilitate immune stimulation or suppress it, thereby influencing the development of inflammatory, autoimmune, and infectious diseases. Supporting evidence from various studies has demonstrated the significant role of EVs in diverse cellular physiological processes, including metabolic disorders and the development of several pathological conditions [62]. Consequently, they hold promise as agents for immune system modulation. EVs can act as a decoy for viruses [67] and bacterial toxins [68]. It is already known that EVs and viruses share common aspects such as their biogenesis, uptake, and ability to carry a specific cargo while being different entities [69,70]. 

### EVs Mediated Cargo Delivery in the Face of SARS-CoV-2 Infection

Recent findings have demonstrated that viruses take advantage of EVs for cellular release, and EVs control viral entry mechanisms for cargo delivery [71,72,73,74]. The viruses use EVs endocytic routes to enter uninfected cells and change the EVs’ secretory pathway to exit infected cells [70] (Figure 1). 

Numerous published reviews have addressed the immunological characteristics of these EVs in the context of viral infections as well as broadly categorized their composition and molecular contents [20,21,70,71]. While different studies suggest that circulating EVs carry SARS-CoV-2 RNA, others have not detected subgenomic RNA fragments that serve as an indicator of an active infection [42,75]. Further studies are therefore underway to evaluate SARS-CoV-2 genomic and subgenomic RNA expression in circulating EVs. Although the studies have indicated that SARS-CoV-2 RNA is detectable in plasma EVs from early infection onward, the form of this EV RNA cargo is unclear. Thus, a thorough understanding of their entry mechanisms is essential for effectively combating pathogenic viruses that exploit EVs for transmission.

## 3. EVs as Potential Role for Immune System Evasion during SARS-CoV-2 Infection

Viruses have developed intricate strategies to counteract the innate immune response, including interfering with antigen presentation, disrupting interferon signaling, and producing “decoy” sub-viral particles that bind to neutralizing antibodies (nAbs), thereby reducing their effective concentration available for neutralizing infectious virions [11,12,13,14,15,16].

The virions consist of a structural spike glycoprotein, an M-membrane protein (a type III transmembrane glycoprotein), an N-nucleocapsid protein (present within the phospholipid bilayer), and non-structural proteins [76,77]. Approximately one-third of the RNA sequence encodes four fundamental structural proteins: spike (S), envelope (E), membrane (M), and nucleocapsid (N) proteins. The remaining two-thirds of the viral genome consists of ORF1a and ORF1b, which encode non-structural replicase/transcriptase proteins [78]. According to recent investigations, the immune escape mutants have appeared and reappeared in chronic COVID-19 patients and immunocompromised individuals who are unable to successfully battle infection, resulting in the major alterations in the SARS-CoV-2 spike as well as the proteins ORF and nsp1 [79].

SARS-CoV-2 enters the host through the respiratory tract and infects the cell mainly via the angiotensin-converting enzyme 2 (ACE2) receptor. ACE2 is a type I integral membrane protein involved in the renin–angiotensin system, which is found in the kidney, testis, intestine, lung, retina, cardiovascular system, adipose tissue, and central nervous system [80]. Once SARS-CoV-2 is internalized into the cell cytoplasm, its lipid bilayers are dismantled by lysosomal enzymes. Subsequently, SARS-CoV-2 utilizes the host cell’s RNA polymerase to replicate its viral single-stranded RNA, thereby increasing the viral load within the host cell [81] (Figure 1). 

The SARS-CoV-2 pathogenicity is highlighted by the entry of the virus through ACE2 receptors, cleavage of the complex, and activation of the S-protein by TMPRSS 2 [82]. According to structural analyses, the spike protein of SARS-CoV (SARS-S) contacts the apex of subunit I of the ACE2 catalytic domain. Once attached to ACE2 by SARS-CoV, the ectodomain of ACE2 is cleaved, which is accompanied by endocytosis of the transmembrane domain into the cell and sometimes internalized as an intact molecule [83]. The internalization and virus particle–host cell fusion is essential for virus entry [84] (Figure 1). 

In the pathogenesis of COVID-19, cells that express ACE2 and CD9 can transfer these viral receptors to other cells via EVs, making recipient cells more susceptible for SARS-CoV-2 infection. Recent investigations have shed light not only on the presence of ACE2 within EVs but also on the capacity of the EVs to transfer ACE2 across various cell types [85]. In this context, SARS-CoV-2 gains access to target cells through binding to exosomal ACE2. However, recent studies have underscored the virus’s strong dependence on endocytic mechanisms with the ability to undergo swift endocytosis in cells that express high levels of ACE2 [86] (Figure 1).

Hence, an alternative mechanism for EV-mediated viral entry involves one of the most highly expressed surface proteins on EVs, tetraspanin CD9 [75]. Research has indicated that CD9 collaborates with TMPRSS2 in cleaving viral fusion glycoproteins, expediting the entry of coronaviruses [74], such as MERS-CoV [87], into lung cells. These findings suggest that CD9 and other tetraspanins on the exosomal surface may serve as mediators in SARS-CoV-2 infection [74] (Figure 1). 

Many factors have been associated with both altered ACE2 expression and COVID-19 severity and progression, including age, sex, ethnicity, medication, and several comorbidities, such as cardiovascular disease and metabolic syndrome. Although ACE2 is widely distributed in various human tissues and many of its determinants have been well recognized, ACE2-expressing organs do not equally participate in COVID-19 pathophysiology, implying that other mechanisms are involved in orchestrating cellular infection, resulting in tissue damage [74]. 

Thus, one of the immune evasion strategies employed by SARS-CoV-2 involves the release of exosomes [88], which can carry viruses, viral proteins, and genetic material. Many studies have suggested the role of released SARS-CoV-2-loaded exosomes and other EVs as potential mechanisms for COVID-19 infection relapse [30,89]. The composition of plasma exosomes varies with the severity of the disease, with mild disease-associated vesicles modulating antigen-specific CD4 T cell responses and severe disease-associated vesicles linked to chronic inflammation [90]. Therefore, analyzing exosome components provides valuable insights into different disease states.

In a recent investigation, it has been revealed that EVs harboring SARS-CoV-2 may contribute to the resurgence of viral RNA in convalescent COVID-19 patients, occurring 7–14 days post-discharge [29]. This indicates the possibility that viral material might have been hidden within these EVs during this ‘inactive’ period and subsequently re-disseminated [29]. Corroborating this, Barberis (2021) demonstrated that RNA from the virus was detected in the exosomal contents of COVID-19 patients’ serum, indicating that the virus can spread through endocytosis [91]. Additionally, the composition of serum-derived exosomes has been found to correlate with disease severity.

The exosomes have been observed to protect viruses from antibody neutralization, facilitating viral integration into cells that typically lack viral receptors [92]. This phenomenon promotes enhanced viral proliferation and renders these cells resistant to pathogens [89]. Moreover, studies have shown an increase in ACE2-expressing extracellular vesicles (evACE2) in the plasma of COVID-19 patients [93], which has been associated with immune response, inflammation, coagulation pathways, and pathology-related clinical indicators [91,94].

**Figure 2:**
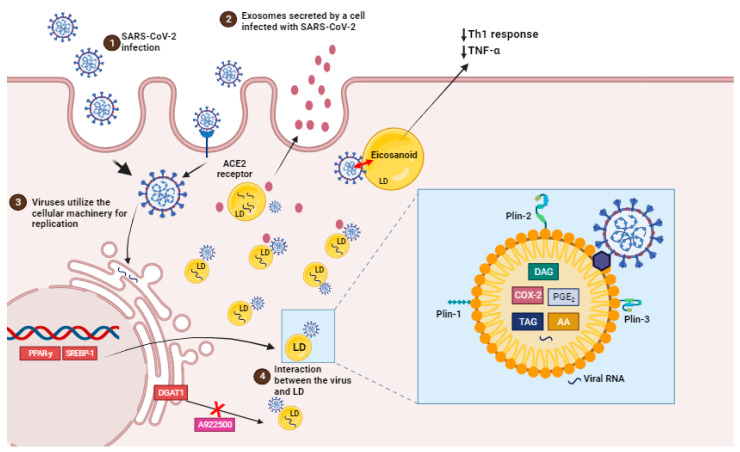
SARS-CoV-2 infection induces lipid droplet formation. (1) SARS-CoV-2 infection, via ACE2 receptor, can increase the activation of important transcription factors, such as SREBP1 and PPAR-gamma, leading to an increased induction of LDs biogenesis and the production of eicosanoids which modulate the host’s immune response. (2) EVs containing viral cargo are released into the extracellular environment. (3) Virus utilie the ER machinery for replication. (4) LDs interact with virus and support SARS-CoV-2 replication (box). Pharmacological inhibition and the genetic knockdown of SREBPs and DGAT1 inhibitor (A922500) can reduce SARS-CoV-2 replication and LD biogenesis. In detail, viral particles interact with LD, which is exhibiting structural proteins, such as PLIN-1, PLIN-2 and PLIN-3, and viral RNA. AA, arachidonic acid; COX-2, cyclooxygenase; DAG, diacylglycerol; PGE2, prostaglandin E2; TAG, triacylglycerol.

The virus co-opts the host’s lipid metabolism to optimize its replication dynamics. By targeting the host cells’ LDs, the principal reservoir of neutral lipids, the SARS-CoV-2 acquires energy substrates crucial for supporting its replication cycles [17] (Figure 2). 

The viral replication process is further augmented through the exploitation of the host cell’s secretory machinery for egress [95]. SARS-CoV-2 virions undergo trafficking to lysosomes and subsequently exploit exocytic lysosomes as a means of egress. In situations where SARS-CoV-2 enters lysosomes via late endosomes or multivesicular bodies (MVBs), it is plausible that the contents of the virus and exosomes encounter each other prior to the extracellular release of exosomes [57,95]. Thus, EVs derived from virus-infected cells have been shown to negatively impact the immune response and facilitate viral proliferation [91].

## 4. Impact of SARS-CoV-2 Infection in the Cellular Lipid Metabolism

Recent data suggest that lipid domains, known as lipid droplets, play an important role in SARS-CoV-2 replication and the synthesis of inflammatory mediators during the disease as well as in other viral infections such as dengue HCV, DENV, and rotavirus. Many viruses replicate their genomes on the surface of phosphatidylethanolamine (PE)/cholesterol-rich organelles. Organelles with pre-existing PE and cholesterol pools are hijacked and further enriched in these lipids. 

The virus’s cellular tropism for lipid metabolism cell machinery suggests that the virus may exploit endogenous lipid materials of different forms, such as lipoproteins and exosomes, as “Trojan horses” to facilitate immune evasion in their systemic spreading [40]. Some evidence also suggests that LDs play a role in virus proliferation and pathogenesis, highlighting the potential of these structures as therapeutic targets [19,96,97,98,99,100,101]. Moreover, LDs accumulation was found in human monocytes from COVID-19 patients as well as in in vitro infection models using human lung epithelial cell line (A549) and human lung microvascular endothelial cell line (HMVEC-L) [17]. 

LDs are intracellular structures that contain triglycerides, cholesterol esters, and enzymes involved in lipid synthesis and storage (Figure 2, box). The expression of proteins linked with lipid metabolism and de novo lipid synthesis is modified during SARS-CoV-2 infection, as well as the pathways involved in lipid uptake, such as CD36 and the primary transcriptional factors involved in lipogenesis, including (peroxisome proliferator-activated receptor) PPAR*γ* and SREBP-1 (Figure 2) [17,102].

SREBP is a transcription factor family that regulates lipid homeostasis by directing the expression of a wide range of fatty acid (SREBP1) and cholesterol (SREBP2) metabolic enzymes [102]. SREBP isoforms have been shown to increase during SARS-CoV-2 infection. Their activation is linked to the immunological response via the increased assembly of the inflammasome complex, which results in the production of IL-1 [103]. Therefore, the authors suggest that the elevated expression and activation of the SREBP pathway are associated with the severity of illness in COVID-19 patients.

Beyond their direct role in viral replication, LDs have also been associated with the establishment of a pro-inflammatory state in COVID-19. In human monocytes infected in vitro with SARS-CoV-2, the accumulation of LDs leads to the upregulation of lipid metabolism-related genes and the release of pro-inflammatory mediators such as leukotrienes (LTB4 and cysLT), chemokines (IL-8 and CXCL10), and inflammatory cytokines (IL-6, TNF, and IL-10). These inflammatory mediators amplify the immune response, leading to the recruitment and activation of immune cells at the site of infection and thereby contributing to COVID-19 development [104,105]. 

In fact, LDs are also sites of compartmentalized synthesis of eicosanoids during inflammatory conditions. Eicosanoids are inflammatory mediators that are derived from the breakdown of arachidonic acid (AA). Phospholipase A2 (PLA2) cleaves LDs and membrane phospholipids to produce AA. AA can be converted into prostaglandins (PGs), thromboxanes (TXs), or leukotrienes (LTs) by cyclooxygenases (COX) or lipoxygenases (LPX), which have regulatory roles in immunological homeostasis. PGE_2_, TXB_2_ and leukotriene B4 (LTB_4_) levels were higher in COVID-19 patients’ bronchoalveolar lavage fluid than in healthy controls. Also, eicosanoid levels were positively correlated with cytokines (IL-1, IL-6, TNF-, IL-12p70, IL-22, and IFN-2) and chemokines (CCL2, CCL11, CXCL9) [18]. PGE_2_, the most prevalent PG, is a key mediator in a variety of physiological processes, particularly the development and regulation of inflammation [106]. SARS-CoV-2 can induce COX-2 overexpression in a variety of human cell lines [18]. Obesity, old age, and a sedentary lifestyle are all risk factors for severe COVID-19, all these risk variables being positively connected with patient serum PGE_2_ levels. According to clinical study, elevated serum PGE_2_ levels in COVID-19 patients are associated with a worsening condition. Increased COX-2 levels were seen in living human precision-cut lung slices, implying a link between ARDS and PGE_2_ prognosis [107].

Leukotrienes are thought to be implicated in COVID-19 patients’ tissues because of increased neutrophil infiltration and neutrophil/lymphocyte ratios [108]. In addition, single-cell examination of COVID-19 patients’ bronchoalveolar immune cells and PBMCs reveals enhanced 5-lipoxygenase expression [109,110]. TNF, IL-1, IL-6, CCL2, and other cytokines are released in the microenvironment by leukotrienes, amplifying inflammatory reactions [111]. A lipidomic study of bronchoalveolar lavage fluid recovered from COVID-19 patients requiring mechanical ventilation revealed significantly higher levels of leukotrienes, primarily LTB_4_, LTE_4_, and eotaxin E4, suggesting leukotrienes’ detrimental effect [112].

Furthermore, some viruses can employ LDs as a crucial site for viral component accumulation, assisting in the production of viral replication complexes [113,114,115,116]. It has been discovered that LDs can be used as platforms for the assembly and maturation of new virus particles just before release. SARS-CoV-2 proteins and ds-RNA are currently tightly connected with the LDs and, in some circumstances, colocalizing with LDs in an in vitro infection model, indicating a probable function for LDs in the SARS-CoV-2 replication cycle [17]. Riccardi et al. found that LDs were near viral non-structural protein NSP6. Additionally, they demonstrated that NSP6 modulates the interaction between LDs and replication organelles-like structures (ROLSs). These authors suggest that the presence of LDs is essential to support the ongoing process of viral replication [117]. 

Similarly, the intimate relationship between LDs and lipid rafts, which are specialized membrane microdomains rich in cholesterol and sphingolipids, may be important for SARS-CoV-2 entry into host cells. LDs might help in the concentration of viral entry receptors such as ACE2 and viral fusion proteins in lipid rafts, thus providing an appropriate environment for virus–cell fusion and internalization. SARS-CoV-2 infection has been shown to promote lipid metabolic reprogramming and LDs formation, which favors virus uptake and the replication conditions [118].

During COVID-19, the role of extracellular vesicles (EVs) in lipid metabolism remained limited. The transmission of viral components, including RNA, from infected to uninfected cells has been related to EVs. This could potentially facilitate the spread of the virus within the host, assisting viral replication and disease progression. Furthermore, EVs containing viral components can either stimulate or depress the immune system, influencing the severity and outcome of COVID-19. 

In parallel, there is evidence that cells can release LDs into the extracellular space, such as LDs being secreted from milk duct cells into milk [119] and LDs moving between epithelial cells in vitro (KB HeLa cells) [120]. It was also recently proposed that LDs could be bundled within adipocyte-derived EVs, activating macrophages in surrounding adipose tissue [121]. There is a strong synergy between these cellular particles based on their similarities and the increasing role of LDs in intercellular communication. Some data suggest a previously unknown overlap and potential interaction between EV and LDs [122]. Collectively, these observations propose that LDs and EVs also contribute significantly to intercellular communication during SARS-CoV-2 infection.

EVs have attracted a lot of focus as possible diagnostic and prognostic biomarkers for SARS-CoV-2 infection. In a study involving the proteome analysis of plasma EVs in COVID-19 patients, it was revealed that 174 proteins exhibited differential expression compared to healthy participants. These proteins are associated with lipid metabolic processes, cellular response, and reactions to stress induced by oxygen-containing substances. The specific lipid profiles of EVs in the bloodstream could provide useful information about disease development and therapy response [123]. 

On the other hand, lipid metabolism and in particular the sphingolipid ceramide is involved in EVs formation [48,49]. Additionally implicated in the ESCRT-independent pathway for EVs production are nSMase and its product ceramide. Ceramide can, in fact, cause EVs to internalize into late endosomes. Ceramides’ unique cone-shaped structure causes spontaneous membrane invagination, which permits the development of intraluminal vesicles into MVBs and the preservation of vesicle shape and structure. A study showed that nSMase inhibition decreases exosome release and that the exosomes were ceramide-enriched [48]. The enzyme acid sphingomyelinase (aSMase) further promotes the production and release of EVs. This acidic enzyme can be activated in response to stimuli such as CD95 or *Pseudomonas aeruginosa* infection as well as in injured cells. Its activation occurs through either direct translocation or lysosomal exocytosis to the cell surface [124,125,126]. Corroborating that, Bianco et al. showed that p38 MAPK activation is necessary for the release of MVs from glial cells, which is triggered by aSMase activity [127].

The composition of EVs is changed by inhibiting neutral SMases (with GW4869 or RNA interference) in two distinct ways: it reduces exosome release while increasing microvesicle budding on the plasma membrane [49]. Lipidomic investigations reveal a distinct lipid composition in the early Golgi, the site of viral budding, consisting of SL-rich lipid nanodomains and localized ordered cholesterol [128]. Furthermore, inclusion of the cellular transmembrane protein Serine Incorporator 5 (SERINC5), which is involved in the synthesis of sphingolipids and phosphatidylserine, into budding virions lowers the infectivity of the virus. This is probably due to the fact that it prevents the entry of SARS-CoV-2 viral-cell fusion, thereby preventing the fusion between the virus and the cell. However, the transmembrane protein ORF7a of SARS-CoV-2 possesses the capability to impede the antiviral function of SERINC5 by hindering the incorporation of overexpressed SERINC5 into budding virions [129].

Several investigations on COVID-19 have revealed that the virus influences lipid metabolism. COVID-19 patients frequently have dysregulated lipid profiles, including elevated levels of triglycerides, cholesterol, and low-density lipoprotein (LDL) cholesterol, all of which are linked to a higher risk of severe consequences [123]. Once within the cell, the virus replicates using the host’s lipid metabolic machinery. Lipids play an important role in the viral envelope, which replaces the viral genetic material and proteins [130]. The endoplasmic reticulum (ER) during the infection is transformed into a site of viral propagation. SARS-CoV-2 causes ER membrane rearrangements, resulting in formations known as “double-membrane vesicles” where viral replication occurs [131]. The ER’s lipid synthesis pathways are co-opted to supply the building blocks for these vesicles and the viral envelope (Figure 2). 

### Modulation of Cellular Lipid Metabolism as Therapeutic Target on SARS-CoV-2 Infection

It has been suggested that medications that interfere with LDs or lipid metabolism can be used as treatments to prevent SARS-CoV-2 infection because of the role that LDs play in viral replication. SARS-CoV-2 alters lipid metabolism in Calu-3 cells by activating the transcription factor SREBP, which promotes lipid remodeling by increasing triglycerides and cholesterol consequently causing LDs accumulation [102]. In addition, double-gene knockdown and SREBP-1 pharmacological inhibition with fatostatin inhibited viral multiplication as well as pro-inflammatory cytokines IL-1 and IL-18. The inhibition of SREBP-1 reduced caspase-1 activation and avoided cell death caused by SARS-CoV-2 infection [102]. These findings suggest that SARS-CoV-2 may exploit the lipid metabolic machinery of host cells to enhance replication. 

Several enzymes control the formation of LDs, including fatty acid synthase (FASN)-producing fatty acids, which are then transformed by diacylglycerol o-acyltransferase 1 and 2 (DGAT1/2) to triacylglycerols. The enzymes acyl-CoA cholesterol acyltransferase 1 and 2 (ACAT1/2) are responsible for cholesterol production in LDs [132,133], thus inhibiting LDs formation with A922500, an inhibitor of the enzyme DGAT-1, and reducing viral replication while protecting cell integrity in infected monocytes [17]. The interaction between LDs and SARS-CoV-2 appears to favor viral replication (Figure 2). The SARS-CoV-2 N protein has been shown to upregulate DGAT1/2 expression to create new LDs. On the LDs’ surface, the N protein interacts with PLIN-2, a structural protein of LDs, to promote viral replication [134]. Fatty acids are also required for the replication of SARS-CoV-2. Exogenously administered fatty acids restored SARS-CoV-2 replication after pharmacological inhibition of fatty acid synthase [135,136]. However, investigations on the precise involvement of fatty acids in SARS-CoV-2 replication are limited.

Understanding the complex link between SARS-CoV-2 and lipid metabolism is important in developing specific therapy options [137]. In this context, statins have a promising future for treating COVID-19 since cellular cholesterol is essential for SARS-CoV-2 viral entrance [138]. In general, a drug that modulates LDs formation, eicosanoids synthesis, and host lipid metabolism holds promise for developing therapeutic strategies. 

In parallel, the development and application of sphingolipid-targeting compounds with dependable and selective qualities may provide new therapeutic options for infectious and other disorders, including COVID-19. Sphingolipids control the replication of viruses by increasing endo-lysosomal Cer, which prevents SARS-CoV-2 from replicating in lysosomal compartments when CDases are inhibited (by using AKS488 or fluoxetine) [139]. Additionally, during SARS-CoV-2 infection, a notable increase in glycosphingolipids was seen, which is a crucial stage for viral replication; treatment with glucosylceramide synthase (GCS) inhibitors reversed this trend [140]. Similarly, it has been shown that important enzymes in the glycerophospholipid metabolism pathway, such as phosphatidic acid phosphatase 1, influence SARS-CoV-2 replication [141].

## 5. EVs as Therapeutic Target

EVs can act as a decoy for viruses [69] and bacterial toxins [68], suggesting a potential role as therapeutic agents. Before the COVID-19 pandemic, some research groups envisioned the use of exosomes as immunogenic components for the treatment of virus infections utilizing models of SARS coronavirus infection. Kuate et al. showed that EVs containing the SARS coronavirus spike S protein caused neutralizing antibody titers that were enhanced by priming with the SARS coronavirus spike vaccine [142]. Thus, coronavirus EVs may be effective for delivering therapeutic substances and inducing immune cell responses in the patient. Drugs or biological modulators that decrease viral propagation and replication in infected cells can be delivered into the EVs [142,143]. EVs have potential advantages, such as cell origin, safety, and consistency, which distinguish them from other delivery techniques such as liposomes [144]. Consequently, inhibiting EVs/MVs absorption by nearby cells could be another beneficial strategy for preventing virus propagation [145]. 

Additionally, mRNAs, microRNAs, and DNA fragments carried by EVs can regulate gene expression in recipient cells [146]. The diverse range of EVs and their cargo underscores the potential of utilizing EVs for therapeutic purposes, as they possess a repertoire of bioactive molecules with a combinatorial capacity that would be challenging to replicate artificially [147]. As a result, miRNAs and lncRNAs extracted from exosomes could serve as biomarkers and potential drug carriers/delivery vehicles while acting as regulators of innate and acquired immunity through the stimulation of cytokine production, inflammatory responses, antigen presentation and lipid mediator synthesis. Thus, we can suggest that EVs have the potential to be used as a therapeutic tool, given their ability to participate in immune modulation and act as delivery vehicles containing various molecules.

Simultaneously, some efforts should be directed to suppressing or removing EVs that selectively promote disorders but are not beneficial, thereby adding novel therapy options to present therapies. Exosome formation and uptake can be inhibited, according to some studies [61,144]. Some researchers are trying to investigate EVs inhibitors as research tools for investigating EVs biology; however, others have assessed such medications’ inhibitory capability in several illness models [143,144]. Furthermore, significant efforts would be required to explore inhibitor effects on exosome release from both healthy and unhealthy cells as well as to create methods to selectively deliver inhibitors to target cells. Selecting a source for obtaining EVs to suppress/improve unpleasant consequences of disease is the gold standard, especially in COVID-19 viral pneumonia. EVs can function as exosome-based nanocarriers for therapeutic agents to target cells.

The source of immunostimulatory exosomes for human antiviral vaccines is critical and must be further investigated [148]. Recently, it was reported that EVs harboring the SARS-CoV-2 spike S-protein enhanced the growth of antibody titers [141]. In mice, an EVs vaccine including the S-protein was evaluated and compared to an adenoviral vector vaccination against SARS-CoV-2. Both types of vaccinations increased antibody titers. When the S-protein containing the EVs vaccine was given to individuals recovering from SARS, antibody titers increased significantly [149]. Recent research has revealed the possibility of developing EV-based vaccinations against COVID-19 induced by SARS-CoV-2. SARS-CoV-2 structural proteins were given as antigens in EVs produced for this new vaccination. This virus-free EVs vaccination against SARS-CoV-2 has the potential to be both effective and safe [150].

EVs, on the other hand, show significant potential in the diagnosis of COVID-19 and the prediction of mild and severe disease progression. SARS-CoV-2 infection produces thrombosis, and disease severity is closely linked with thrombosis development. Some clinical investigations show a large rise in circulating EVs carrying tissue factor (TF)/CD142 levels in COVID-19 patients as well as a strong connection with thrombosis, malignant disease development, and hospitalization length [151].

Additionally, EVs containing ACE-2 can be employed as SARS-CoV-2 neutralization traps. Experiments with high-speed atomic force microscopy revealed that EVs secreted by the ACE-2 expressing cell line PC9 bind directly to SARS-CoV-2 particles and initiate the membrane docking mechanism via ACE-2 interaction, implying that ACE2-EVs could be used as neutralization traps for SARS-CoV-2 infection. In vitro, ACE2-EVs from COVID-19 patients’ plasma and ACE2-EVs released by the ACE-2 expressing human lung epithelial cell line Calu-3 inhibit SARS-CoV-2 and VOC infections with greater efficacy than soluble ACE-2. Both human lung spheroids cell-derived ACE2-EVs and plasma-derived ACE2-EVs from patients with COVID-19 delivered via inhalation therapy promote viral clearance and reduce lung injury in cynomolgus macaques and hACE-2 transgenic mice challenged with authentic SARS-CoV-2, suggesting a potential function for EVs in the SARS-CoV-2 infection control mechanism [36,152]. 

Hence, EVs find application as natural antigen carriers and for directing therapies toward distant organs. EVs can be used either in their naturally secreted state or in an engineered form for tailored therapeutic purposes [153,154,155]. While EVs are excellent as carriers for viral antigens, presenting them in their unaltered form to evoke an effective immune response, they also have the capacity to transport host-derived antiviral compounds and immune enhancers [156,157]. Notwithstanding, the challenges associated with employing EVs as therapeutics, their favorable biological attributes and natural carrier capabilities for small molecules render them a compelling choice for vaccine development. Current EVs isolation and purification techniques can be harnessed with the utmost potential to adhere to cGMP requirements. Both the International Society for Cellular and Gene Therapies and the International Society for EVs have recently issued a statement advocating for the development of EV-based therapeutics derived from MSCs and other cells for COVID-19 treatment but under strict regulatory oversight [158].

It is vital to highlight that the role of extracellular vesicles varies depending on the virus, infected cell type, and infection stage. Understanding the roles of EVs in virus infections provides essential knowledge into the complicated interactions between viruses and their host cells and may lead to the development of new antiviral approaches and therapies.

## 6. Conclusions

The battle between viruses and their hosts has a long history. Whereas the immune system has evolved to protect from these pathogens, viruses have acquired clever evasion molecular mechanisms to avoid being detected and destroyed by the immune system. Given their extraordinarily high transmission rates and the possibility of recurrent outbreaks, there is an urgent need to find effective novel antiviral medicines for both treating present respiratory virus infections and preventing future outbreaks.

COVID-19 patients usually have dysregulated lipid profiles, which is associated with an adverse outcome of disease. As the virus is used to provide the building blocks for these vesicles and the viral envelope, they have developed sophisticated evasion strategies that explore lipid metabolism to replicate and avoid being recognized and destroyed by the immune system. Exosomes and respiratory viruses, such as coronavirus and other viruses, appear to make use of sorting complexes and cellular mechanisms that contribute to either maintain host homeostasis or allow viral replication, despite their distinct evolutionary origins. As a result, the development of exosome-based treatments, which include natural exosomes, nano-decoys, and antiviral-loaded exosomes, provide a range of treatment optimization choices that can successfully target, bind to, and limit the cellular absorption of various viruses. Thus, exosomes present great promise as natural therapies and new drug delivery vehicles due to their intrinsic involvement in intercellular communication as well as their innate immunogenic and cytotoxic capabilities.
